# Structured antenatal milk expression education for nulliparous pregnant people: results of a pilot, randomized controlled trial in the United States

**DOI:** 10.1186/s13006-022-00491-8

**Published:** 2022-07-07

**Authors:** Jill R. Demirci, Melissa Glasser, Katherine P. Himes, Susan M. Sereika

**Affiliations:** 1 Department of Health Promotion & Development, University of Pittsburgh, Pittsburgh, PA USA; 2 Department of Obstetrics, Gynecology, and Reproductive Sciences, Division of Maternal-Fetal Medicine, University of Pittsburgh, Pittsburgh, PA USA; 3 UPMC Magee-Womens Hospital, Pittsburgh, PA USA; 4 Department of Health & Community Systems, University of Pittsburgh, Pittsburgh, PA USA

**Keywords:** Breastfeeding, Human milk, Milk expression, Pregnancy, Antenatal colostrum expression, Antenatal milk expression

## Abstract

**Background:**

Hand-expression, collection, and storage of breast milk during pregnancy (i.e., antenatal milk expression or AME) is a safe, potentially effective practice to reduce early, undesired infant formula supplementation among women with diabetes. The feasibility and potential impact of AME on lactation outcomes in the United States (U.S.) and among non-diabetic birthing people is unknown.

**Methods:**

The purpose of this study was to examine the feasibility of a structured AME intervention among nulliparous birthing people in the United States. We recruited 45 low-risk, nulliparous individuals at 34–36^6/7^ weeks of gestation from a hospital-based midwife practice. Participants were randomized to AME or a control group receiving lactation education handouts. Interventions were delivered at weekly visits at 37–40 weeks of pregnancy. The AME intervention involved technique demonstration and feedback from a lactation consultant and daily independent practice. Lactation outcomes were assessed during the postpartum hospitalization, 1–2 weeks postpartum, and 3–4 months postpartum.

**Results:**

Between December 2016 and February 2018, 63 individuals were approached and screened for eligibility, and 45 enrolled into the study (71%). Of 22 participants assigned to AME, 18 completed at least one AME study visit. Participants reported practicing AME on at least 60% of days prior to their infant’s birth. Most were able to express milk antenatally (15/18), more than half collected and froze antenatal milk (11/18), and 39% (7/18) supplemented their infants with antenatal milk after birth. No major problems were reported with AME. Perinatal and lactation outcomes, including infant gestational age at birth, neonatal intensive care unit admissions, delayed onset of lactogenesis II, and use of infant formula were similar between AME and control groups. Among participants in both groups who were feeding any breast milk at each assessment, breastfeeding self-efficacy increased and perceptions of insufficient milk decreased over the postpartum course.

**Conclusions:**

In a small group of nulliparous birthing people in the U.S., AME education and independent practice beginning at 37 weeks of pregnancy was feasible. In some cases, AME provided a back-up supply of milk when supplementation was indicated or desired. The relationship between AME and lactation outcomes requires further study with adequately powered samples.

**Trial Registration:**

This trial was retrospectively registered at ClinicalTrials.gov on May 11, 2021 under the following registration ID: NCT04929301. https://clinicaltrials.gov/ct2/show/NCT04929301.

## Background

Direct chest/breastfeeding and/or provision of one’s own milk (hereafter, collectively referred to as “breastfeeding” unless otherwise indicated) is considered the biologically normative method to feed infants and young children, with dose-dependent health implications for lactating parents and their children [1]. Although most pregnant individuals in the United States (U.S.) intend to breastfeed after birth, only 30–45% meet their goals for breastfeeding duration and exclusivity [2, 3]. According to the Centers for Disease Control and Prevention, over 80% of U.S. infants begin life breastfeeding, but by two days postpartum, 19% of breastfed infants receive formula supplementation. By 3 months, only 46% of infants are exclusively breastfeeding. By 6 months, only 26% of infants are exclusively breastfeeding, and 57% are breastfeeding at all [4]. Primiparous individuals and those without prior breastfeeding experience appear to be particularly at risk for not meeting their prenatal breastfeeding intentions [3, 5, 6] and a constellation of interrelated lactation problems. Some of these problems include unintended in-hospital infant formula supplementation [7–9], low breastfeeding self-efficacy [10, 11], delayed onset of lactogenesis II (copious milk production following birth) [12, 13], and perception of insufficient milk [11, 14].

Recently, interest has grown in the practice of antenatal milk expression (AME), which may partly address some of these lactation challenges. AME involves hand expression of colostrum in pregnancy, usually commencing between 36 and 37 weeks of gestation. Any colostrum expressed may be collected frozen for later use [15]. AME is sometimes integrated into prenatal education in countries like Australia [16] and the UK [17]. Particularly among nulliparous and diabetic pregnant people, AME may contribute to increased breastfeeding confidence [18, 19] and has been linked to reduced infant formula supplementation during the postpartum hospitalization [20, 21].

Conversely, researchers have also found that AME can cause frustration, embarrassment, and anxiety—particularly when there is difficulty expressing milk [15, 18]. Another common concern about AME is that potential nipple stimulation may increase endogenous circulating oxytocin, contributing to uterine irritability and onset of labor. Yet the largest study of AME to date—the DAME Trial, provided evidence of AME’s safety. The DAME Trial involved 635 women with gestational or preexisting diabetes at low risk for other perinatal complications, 319 of whom were randomized to practice AME twice daily beginning at 36 weeks of pregnancy. The study team found that AME did not influence infant gestational age or neonatal intensive care unit (NICU) admissions, nor was it associated with uterine hyperstimulation or fetal compromise [20].

The majority of the research on AME, including its feasibility, acceptability, and effect on lactation outcomes, has been conducted outside the U.S. and among women with diabetes, whose infants are at risk for formula supplementation after birth due to hypoglycemia [15]. In this paper, we describe the implementation of a structured AME intervention among nulliparous, pregnant people in the U.S. without diabetes, who are also at risk suboptimal lactation outcomes, in the context of a pilot randomized controlled trial. We evaluate feasibility related to recruitment, retention, and delivery/uptake of the AME intervention and describe lactation outcomes across groups.

## Methods

Gender of participants was not explicitly assessed in this study. Thus, gender inclusive terms are used throughout to account for participants in this study who may not have identified as a “woman” or “mother.” When describing infant feeding, we also attempt to use the most specific, gender inclusive descriptors. Sometimes this was precluded, as surveys options were designed with gendered or non-specific terms (e.g., “breast milk” vs. “my own milk”). When referencing others’ research or work, we use their stated terminology.

### Design

In this pilot randomized controlled trial, we recruited nulliparous people in their third pregnancy trimester and used sealed envelope block randomization to assign them to a structured AME intervention or an education control. Participants completed an enrollment/baseline study visit between 34^0/7^ and 36^6/7^ weeks of pregnancy. Study visits to deliver the assigned intervention were scheduled weekly from 37 to 40^6/7^ weeks of pregnancy or until the infant’s birth, whichever occurred first (maximum of four visits in pregnancy). Visits were scheduled based on participant preference—often immediately following a prenatal visit in a clinical research suite adjacent to the prenatal practice. In the postpartum period, we met with participants to administer surveys during the birth hospitalization (1–4 days postpartum), at 1–2 weeks postpartum, and 3–4 months postpartum. Measured outcomes of interest at postpartum follow-ups included breastfeeding self-efficacy, perceived milk supply, perceived onset of lactogenesis II (copious milk production), and infant feeding status/formula use. No formal sample size estimation in terms of testing hypotheses was conducted; instead, the target sample size (*n* = 45) was based on a predefined timeframe and budget that would generate feasibility data for a larger trial. Participants were compensated up to $50 for their participation, based on number of study visits completed.

### Participants and setting

We recruited participants between December 2016 and February 2018 from a hospital-based midwife practice at UPMC Magee-Womens Hospital (Pittsburgh, Pennsylvania). Our primary recruitment strategy was electronic health record review of select eligibility criteria (parity, gestational age), followed by in-person approach of potentially eligible patients at a prenatal visit between 34 and 36^6/7^ weeks of pregnancy to assess interest in study participation. Study flyers with contact information were also posted in the midwife office for patient self-referral.

Interested patients were screened for full eligibility, provided written informed consent, and randomized prior to completing baseline study measures. Eligible participants were at least 18 years old, nulliparous, pregnant with a single fetus, planned to exclusively breastfeed/provide their own milk to their infant for the first four months postpartum, did not have any known physiologic risk factors for insufficient milk supply (e.g., breast hypoplasia, polycystic ovarian syndrome, diabetes, breast reduction surgery), and did not have any conditions constituting a high-risk pregnancy (e.g., vaginal bleeding after the first trimester, fetal congenital anomalies, polyhydramnios, current smoking) [22].

### AME intervention

At an introductory visit to AME during week 37 of pregnancy, participants viewed a video modeling hand-expression of milk [23]. This video exemplar was chosen because it featured close-up footage of a model self-expressing milk using similar techniques advised by the study IBCLC during participant individual instruction (e.g., breast massage prior to and during expression, “c” or “u” shape finger placement back from the nipple, 3-step Marmet technique [24], rhythmic pace while alternating between breasts). Because the model in the video was several days postpartum, participants were cautioned that the volume and appearance of any milk expressed would likely differ.

Following the video, participants were invited to engage in hands-on, guided practice of AME beginning with breast massage and utilizing the Marmet technique [24] with an International Board Certified Lactation Consultant (IBCLC; author JRD). After instruction/practice at the initial visit, we provided participants written and verbal instructions for safe expression, collection, and storage of antenatal milk in the home setting. At subsequent weekly study visits during pregnancy, participants met with the lactation consultant to reinforce AME technique, address questions about AME or breastfeeding, and collect a milk sample if possible for later analyses of macronutrient and immunological composition of antenatal milk (milk samples were also collected from participants in both groups at each postpartum follow-up for comparison). Similar to the protocol for home milk expression described by Forster and colleagues [25], participants were instructed to engage in at-home milk expression and collection one to two times per day for up to ten minutes and record this in a written diary, which was collected at subsequent study visits. Participants were provided sterile, flip-top containers (11 mL Snappies® colostrum collectors) in which to collect and freeze antenatal milk and instructions on how to transport and store antenatal milk at the birth hospital.

### Education control

Participants in the education control group met with study staff during study visits in pregnancy to receive handouts from Lactation Education Resources [26]. Handouts addressed a new theme each week pertaining to breastfeeding preparation and prevention of common lactation problems (Week 37: “Sore Nipples”; Week 38: “Five Keys to Successful Breastfeeding”; Week 39: “Signs of a Good Feeding” and “Is my Baby Getting Enough?”; Week 40: “I wish someone had told me …”). Control group participants did not receive any education on AME or additional lactation education from study staff. Handouts did not address AME. The rationale for offering handouts to the control group was to provide contact episodes with study staff similar in frequency to the AME group and to minimize attrition by offering an educational incentive.

Participants assigned to AME were not provided the hand-outs that the control group received. Participants in both groups may have received lactation education outside of the study, as we did limit or replace any education offered by the prenatal practice, other care providers, or community-based resources.

### Data collection and measurement

At enrollment, participants completed a survey assessing demographics, obstetric and medical history, and breastfeeding attitude (Iowa Infant Feeding Attitude Scale; score range 17–85, with higher scores indicative of more favorable attitude toward breastfeeding) [27]. Breastfeeding intentions/plans were assessed at enrollment with two questions, “After your baby is born, how long do you plan to exclusively breastfeed (i.e., feed baby ONLY breast milk with no formula or other foods)?” and “ After your baby is born, how long do you plan to continue ANY breastfeeding or breast milk feeds?”. Both questions were multiple choice with by-month groupings (e.g., < 6 months) and an “unsure” option.

At enrollment and postpartum visits, surveys included an assessment of prenatal and postpartum breastfeeding self-efficacy, respectively (Breastfeeding Self-Efficacy Scale-SF; score range 14–70 with higher scores indicative of higher self-efficacy) [28, 29]. At enrollment and postpartum visits, we also administered a combined measure of stress, anxiety, and depression, as important correlates of lactation outcomes (Perceived Stress Scale-4 [30], PROMIS Emotional Distress-Anxiety 4-item bank [31], 3-item Pregnancy Risk Assessment Monitoring System (PRAMS) Depression/Anxiety [32]; score range 11–55 with higher scores indicative of higher levels of anxiety, stress, and depressive symptoms).

In a daily written diary, participants in the AME group tracked number and duration of daily home AME sessions, approximate volume of AME milk collected via visual estimation (using 5 mL line on container), and any problems experienced with AME. The latter was assessed via open-ended questions, “Any cramping or side effects during or after expressing?” and “Additional comments?”. At each study visit for AME participants, we recorded volume of milk expressed, problems experienced during AME at that particular visit and as verbalized by the participant (checklist of common concerns/issues previously reported about AME [33] and a free-text field), and whether the participant had experienced any of the following during or directly after AME since the prior study visit: prolonged uterine tightening lasting longer than 1 min, frequent uterine tightening (> 5 times in 10 min), vaginal bleeding, or reduced fetal movement.

We collected infant feeding data from the electronic health record (EHR) during the birth hospitalization, including initiation of direct chest/breastfeeding (any and timing post-birth), and receipt and total volume of expressed milk and infant formula. Additional data collected from the EHR included pregnancy complications and characteristics of the labor and birth. Data were abstracted independently by two researchers and any discrepancies resolved through re-review of EHR data and discussion with the first author.

Postpartum surveys assessed perception of insufficient milk using two methods: 1) a single investigator-created item asking “Do you feel you make enough breast milk to satisfy your baby?” with answer options of “yes,” “no,” or “unsure” (dichotomized for analysis to yes = “no perceived insufficient milk” and no/unsure = “perceived insufficient milk”); and 2) score on the Perceived Infant Breastfeeding Satiety subscale (5 items total) within the H&H Lactation Scale [34]; possible scores ranged from 0–35, wherein lower scores represent lower confidence that one is making enough milk. Infant Satiety subscale scores at one week postpartum have demonstrated predictive validity with breastfeeding continuation at eight weeks. The subscale also exhibited concurrent validity with perception of insufficient milk at eight weeks [34].

Postpartum surveys also assessed current infant feeding status with the following survey item: “How are you currently feeding your baby?” Answer options included “breast milk only,” “formula only,” and “both formula and breast milk.” While it was unlikely that any study infants were receiving pasteurized donor human milk (based on eligibility criteria within the health system), it is a possibility that infants were receiving milk from another lactating parent (i.e., informally shared milk).

Whether any antenatally-expressed milk had been fed to the infant was assessed in each postpartum survey with a dichotomous survey item: “Since your baby was born, have you given him/her any milk that was hand-expressed while you were still pregnant?”. A follow-up free-text item asked participants to estimate the total volume of antenatal milk in milliliters that they had fed to their infant since birth.

The 1–2 week postpartum survey assessed onset of lactogenesis II in days postpartum by asking, “How long did it take for your milk to come in after your baby was born (i.e., when did you notice a big increase in the amount of milk)?”, with the following answer options: 1 day or less, 2 days, 3 days, 4 days, more than 4 days, my milk never came in, and I don't remember when my milk came in. Answer options were then collapsed to < 4 days (normal onset) or ≥ 4 days (delayed onset), which corresponds with research indicating an average onset of lactogenesis II between 50–73 h, with delayed lactogenesis typically classified as > 72 h postpartum [35, 36]. No participants selected that their milk never came in. One participant did not remember when their milk came in and was not included in descriptive statistics for this variable. The language of the question was adapted from a validated two-question assessment of lactogenesis II based on maternal perception, which demonstrated high sensitivity and specificity for detecting delayed lactogenesis II compared to the gold standard of infant test weights [37]. However, the validated assessment queried postpartum individuals three times daily, beginning at 24-h postpartum and recorded responses to the nearest hour. Our adaptation to timing the assessment at 1–2 weeks postpartum was intended to minimize cumulative participant survey burden in the postpartum period. In addition, our answer options were adapted from a by-hour recall to a by-day recall, based anticipated difficulty for participants in recalling details at 1–2 weeks postpartum.

Qualitative interviews were conducted with participants assigned to AME to explore their study experiences and assess acceptability of the intervention. Those results are published elsewhere [19].

### Analysis

Feasibility of recruitment and retention was assessed via screen-to-consent ratios, attrition rates, and incidence of unintentional intervention cross-over. We calculated descriptive statistics for sample characteristics, uptake of the AME intervention (frequency of expressing episodes, completion of study visits), problems with AME, and lactation and perinatal health outcomes using SPSS v. 27 (IBM Corporation, 2020).

## Results

### Sample characteristics

**Fig. 1:**
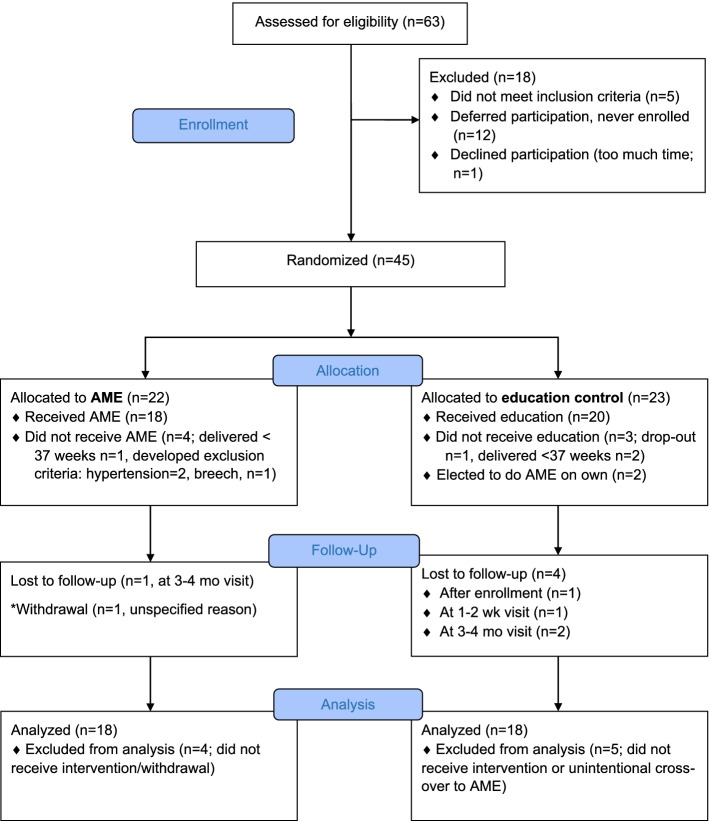
Consort flow diagram. *withdrawal also did not receive intervention, withdrawal occurred after postpartum hospital study visit

Of 63 people assessed for eligibility, 45 (71%) met inclusion criteria, consented to study participation, and were randomized and enrolled in the study over a 14-month period (AME: *n* = 22; Control: *n* = 23). Two control group participants who were IBCLCs notified study staff that they were engaging in AME on their own during the study period. These participants were dropped from the analysis. One participant in the AME group was also an IBCLC. Thirty-six participants completed at least one study visit for their assigned group without intervention crossover (AME: *n* = 18; Control: *n* = 18) and were included in analyses (Fig. 1). Most participants were white, married, possessed at least a Bachelor’s degree, and planned to exclusively breastfeed to at least six months postpartum. Participants assigned to the AME and control groups were comparable in terms of demographics, breastfeeding plans, breastfeeding attitudes, and other characteristics at enrollment (Table 1).

**Table 1:** Participant characteristics at enrollment (34–36^6/7^ gestational weeks)

	AME (*n* = 18)	Control (*n* = 18)
Married [n (%)]	14 (78)	13 (72)
Education [n (%)]		
*High school or less*	0 (0)	0 (0)
*Associate’s or vocational degree/some college*	1 (6)	3 (17)
*Bachelor’s degree*	9 (50)	9 (50)
*Post-graduate degree*	8 (44)	6 (33)
Race [n (%)]		
*White/Caucasian*	17 (94)	15 (83)
*Black/African American*	0 (0)	2 (11)
*Other*	1 (6)	1 (6)
Hispanic ethnicity [n (%)]	1 (6)	0 (0)
WIC^a^ recipient [n (%)]	0 (0)	2 (11)
Employed (at enrollment) [n (%)]	18 (100)	14 (78)
Age [median (IQR)]	31.5 (4.5)	31.0 (7.5)
Pre-pregnancy BMI [median (IQR)]	23.6 (3.6)	22.6 (5.0)
Planned duration of any breastfeeding [n (%)]		
*Unsure*	4 (22)	2 (11)
≤ *6 months*	0 (0)	0 (0)
> *6 months*	14 (78)	16 (89)
Planned duration of exclusive breastfeeding [n (%)]		
*Unsure*	3 (17)	2 (11)
< *6 months*	2 (11)	3 (17)
≥ *6 months*	13 (72)	13 (72)
Endorsed breast growth in pregnancy [n (%)]	15 (83)	17 (94)
BSES-SF score at enrollment^b^ [median (IQR)]	53.0 (6.0)	42.0 (15.0)
IIFAS score at enrollment^c^ [median (IQR)]	69.5 (7.3)	62.0 (8.0)
EDA/PSS score at enrollment^d^ [median (IQR)]	20.0 (8.3)	23.0 (7.3)

^a^WIC: Special Supplemental Nutrition Program for Women, Infants, and Children, a U.S. federal assistance program serving low-income families

^b^BSES-SF: Breastfeeding Self-Efficacy Scale: higher score indicative of greater breastfeeding self-efficacy

^c^IIFAS: Iowa Infant Feeding Attitude Scale: higher score indicative of more positive breastfeeding attitude; *n* = 17 for control group

^d^EDA/PSS: combined PROMIS Emotional Distress Anxiety 4-item bank, Perceived Stress Scale-4 item and PRAMS Depression/Anxiety 3-item, higher score indicative of greater depression/anxiety

### AME uptake and problems/concerns

Of the 18 participants who received the AME intervention, 5 (28%) completed all four weekly study visits before their infant’s birth. For participants who completed written AME diaries, most reported that they practiced AME at least once each day (37 weeks: *n* = 12/15 (80%); 38 weeks: *n* = 11/18 (61%); 39 weeks: *n* = 10/14 (71%); 40 weeks: *n* = 7/7 (100%)). All participants reported practicing AME on at least 60% of all days between the initial AME study visit and their infant’s birth. Sixteen (89%) reported practicing AME on at least 80% of all days.

**Fig. 2:**
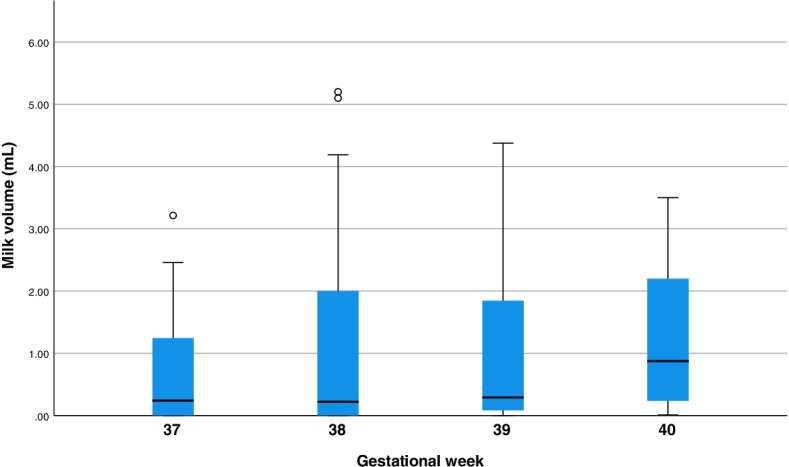
Box and whisker plot of average individual milk volume (mL) expressed per AME episode from gestational weeks 37 to 40 (37 weeks: *n* = 15, 38 weeks: *n* = 18, 39 weeks: *n* = 14, 40 weeks: *n* = 7). Horizontal lines in shaded area represent medians across individual participant averages

Most participants (*n* = 15/18) were able to visualize milk during AME, 61% (*n* = 11/18) froze any antenatal milk, and 39% (*n* = 7/18) fed any antenatal milk to their infant after birth (*n* = 5 during the postpartum hospitalization, *n* = 4 in the first two weeks postpartum). Reasons for feeding AME milk postpartum have previously been described [19], but briefly they included parental or hospital staff perception of inadequate milk transfer during direct chest/breastfeeding and unavailability of the participant for direct chest/breastfeeding due to medical complications at birth (e.g., preeclampsia). Volume of milk expressed/collected during AME episodes varied considerably among participants, with a median total of 5.8 mL per participant over the study course. Median volumes of antenatal milk expressed/collected per AME episode slightly increased from 37 to 40 weeks: 37 weeks: 0.24 mL; 38 weeks: 0.22 mL; 39 weeks: 0.29 mL; 40 weeks: 0.88 mL (Table 2; Fig. 2), though there were fewer participants with milk volume data during the 40^th^ gestational week (*n* = 7), compared to other weeks (*n* ≥ 14).

**Table 2:** AME intervention uptake from 37^th^ week of pregnancy until infant’s birth (*n* = 18)

Characteristic	Median (IQR); range
Number of AME study visits	3.0 (2.0); 1–4
Total times practicing AME independently	15.5 (14.3); 0–45
Total milk volume collected during AME (mLs)	5.8 (45.2); 0–93.3

No participants reported experiencing decreased fetal movement, frequent or prolonged uterine tightening, or vaginal bleeding during or directly following AME. Almost three-quarters of participants (*n* = 13/18) reported minor problems or concerns during or directly following AME, including: transient uterine tightening, pressure, or cramping (*n* = 11); increased fetal movement (*n* = 5); breast discomfort (*n* = 2); breast skin irritation (*n* = 1); fatigue in the hand or arm expressing (*n* = 2); nausea (*n* = 1); difficulty collecting milk due to problems visualizing placement of collection container under breasts (*n* = 1); and expressing less milk than the previous week (*n* = 1). There were high rates of obstetric and neonatal complications across both groups, with 36% of the sample (13/36; AME: *n* = 8, Control: *n* = 5) experiencing pre-eclampsia, chorioamnionitis, and/or a NICU admission. Infant gestational age and birthweight appeared comparable between groups (Table 3).

**Table 3:** Perinatal and lactation outcomes

	AME (*n* = 18)	Control (*n* = 18)
Pregnancy complications [n (%)]		
*Preeclampsia*	4 (22)	1 (6)
*Chorioamnionitis (suspected or confirmed)*	3 (17)	2 (11)
NICU admission (birth hospitalization) [n (%)]	6 (33)	4 (22)
Infant gestational age (weeks) [median (IQR)]	40^2/7^ (1^5/7^)	40^1/7^ (1^5/7^)
Infant birthweight (grams) [median (IQR)]	3420 (629.5)	3267.5 (421.3)
Delivery method [n (%)]		
*Spontaneous vaginal*	10 (56)	13 (72)
*Assisted vaginal*	1 (6)	1 (6)
*Cesarean section*	7 (39)	4 (22)
Direct chest/breastfeeding initiation ≤ 1 h after birth [n (%)]	7 (39)	8 (44)
Infant formula in hospital [n (%)]	8 (44)	6 (33)
*Number infant formula feeds [median (IQR)]*^*a*^	3.5 (8.8)	6.5 (13.3)
*Total volume infant formula (mL) [median (IQR)]*^*a*^	76.0 (272.9)	106.3 (601.6)
Expressed milk feeds in hospital [n (%)]^b^	5 (28)	7 (39)
*Number expressed milk feeds [median (IQR)]*^*c*^	2.0 (11.0)	3.0 (3.0)
*Total volume expressed milk feeds (mL) [median (IQR)]*^*c*^	33.0 (367.5)	35.0 (52.5)
Infant feeding status at 1–2 weeks postpartum [n (%)]^d^		
*Only breast milk*	16 (89)	13 (76)
*Breast milk* + *formula*	2 (11)	4 (24)
*Only formula*	0 (0)	0 (0)
Infant feeding status at 3–4 months postpartum [n (%)]^e^		
*Only breast milk*	14 (78)	11 (69)
*Breast milk* + *formula*	1 (6)	4 (25)
*Only formula*	3 (17)	1 (6)
BSES-SF scores [median (IQR)]		
*Postpartum hospitalization*	56 (13.5)	48.5 (17.8)
*1–2 weeks postpartum*^*f*^	59.0 (14.0)	54.0 (17.5)
*3–4 months postpartum*^*g*^	67.0 (4.0)	57.0 (11.0)
H & H Lactation Scale scores (perception of insufficient milk, continuous) [median (IQR)]		
*Postpartum hospitalization*	33.0 (4.0)	31.0 (5.3)
*1–2 weeks postpartum*^*h*^	34.5 (2.0)	32.0 (8.5)
*3–4 months postpartum*^*i*^	35.0 (1.0)	33.0 (7.0)
Perception of insufficient milk (dichotomous) [n (%)]		
*Postpartum hospitalization*	4 (22)	11 (61)
*1–2 weeks postpartum*^*h*^	3 (17)	6 (35)
*3–4 months postpartum*^*i*^	1 (7)	2 (13)
Delayed lactogenesis II [n (%)]^j^	8 (44)	7 (41)

BSES-SF: Breastfeeding Self-Efficacy Scale Short Form, where higher score reflective of greater breastfeeding self-efficacy, range of possible scores 14–70; H & H Lactation Scale scores (PIBSS subscale), continuous-type measure of perceived insufficient milk, wherein lower scores represented lower confidence or perception that one was making enough milk, range of possible scores 0–35; Perceived insufficient milk, dichotomous-type measure, as indicated by “no” or “unsure” response on survey item, “Do you feel you make enough breast milk to satisfy your baby?”; Delayed lactogenesis II defined as onset ≥ 4 days postpartum and assessed via participant recall at 1–2 weeks postpartum; “breast milk” under feed type reflects survey wording provided to participants

^a^Number and volume of in-hospital infant formula feeds, amongst participants whose infants received formula in hospital: Total: *n* = 14, AME: *n* = 8, Control: *n* = 6

^b^Not specified if expressed milk feeds were antenatal or postpartum milk

^c^Number and volume of expressed milk feeds in hospital, amongst participants who infants received expressed milk in hospital: Total: *n* = 12, AME: *n* = 5, Control: *n* = 7

^d^Feeding status at 1–2 weeks postpartum: Total: *n* = 35, AME: *n* = 18, Control: *n* = 17

^e^Feeding status at 3–4 months postpartum: Total: *n* = 34, AME: *n* = 18, Control: *n* = 16

^f^BSES-SF scores at 1–2 weeks postpartum: Total: *n* = 34, AME: *n* = 17, Control: *n* = 17

^g^BSES-SF scores at 3–4 months postpartum: Total: *n* = 28, AME: *n* = 15, Control: *n* = 13

^h^H&H Lactation Scale scores and dichotomous measure of perceived insufficient milk at 1–2 weeks postpartum, amongst participants feeding any breast milk: Total: *n* = 35, AME: *n* = 18, Control: *n* = 17

^I^H&H Lactation Scale scores and dichotomous measure of insufficient milk at 3–4 weeks postpartum, amongst participants feeding any breast milk: Total: *n* = 30, AME: *n* = 15, Control: *n* = 15

^j^Delayed lactogenesis, as assessed at 1–2 weeks postpartum: Total: *n* = 34, AME: *n* = 18, Control: *n* = 16; among the two participants not included, one did not have data for this variable/did not complete 1–2 week survey, and one did not remember when their milk came in

### Lactation outcomes

During the postpartum hospitalization, all participants initiated direct chest/breastfeeding, though more than half supplemented direct chest/breastfeeding with their own expressed milk and/or formula (*n* = 19/36; AME: *n* = 10, Control: *n* = 9). Rates of exclusive breast milk feeding were lowest during the postpartum hospitalization (61%; *n* = 22/36) and highest at the 1–2 week postpartum assessment (83%; *n* = 29/35). Four participants stopped breast milk feeds by the 3–4 month assessment. Infant feeding patterns were similar between groups (Table 3).

Among participants in both groups providing any breast milk, BSES-SF and H & H Lactation Scale scores increased over the postpartum course, indicative of higher breastfeeding self-efficacy and reduced perception of insufficient milk, respectively. The dichotomous-type perceived insufficient milk assessment mirrored the continuous-type measurement (H&H Lactation Scale), with progressively fewer participants over the postpartum course endorsing uncertainty or affirmation that they were not making enough milk, among those still providing breast milk. While nearly half of the sample experienced delayed lactogenesis II (onset of copious milk production ≥ 4 days after birth), the rate of occurrence in both the AME and control groups was similar (Table 3).

## Discussion

In this pilot randomized trial involving a sample of nulliparous birthing people in the U.S., we found that a structured AME protocol involving hand expression 1–2 times per day and weekly reinforcement visits with a lactation consultant beginning at 37 weeks of pregnancy was feasible. Most of those screened were enrolled (~ 71%). There was a moderate rate (20%) of attrition (withdrawals, drop-outs), unintentional intervention cross-over, and intervention non-receipt (development of exclusion criteria, birth prior to 37 gestational weeks). There was high compliance with daily AME, and most participants visualized and collected antenatal milk. Transient uterine tightening/cramping was common during or following AME, but there was no evidence of fetal, infant, or birthing/lactating parent harm associated with AME.

The average volume of milk expressed over the course of study participation was similar to that reported in the DAME Trial, a large randomized trial of AME among women with gestational or preexisting diabetes. Among 241 DAME participants with diabetes assigned to receive AME education who had known expressed milk volumes, the median volume of milk expressed from 36 weeks of pregnancy to birth over a median of 20 expressing episodes was 5.5 mL [20]. In our study, participants expressed a median of 5.8 mL over 15.5 expressing episodes. Similar to the small increase in milk volume per expression episode we observed over progressive weeks of pregnancy, Rietveld (2011) also found milk volume increased over gestational weeks 35–38 among 11 participants with diabetes engaging in AME [38].

We found no evidence that AME was related to adverse safety outcomes, including uterine hypercontractility or onset of labor. NICU admissions, gestational age at birth, infant birthweight, cesarean sections, and vaginal births involving forceps or vacuum assist were proportionally similar between AME and control groups. The most common side effects or problems with AME in our sample were transient uterine contractility and increased fetal activity during or directly following AME. This aligns with the findings of the DAME trial; their study team conducted cardiotocography surveillance during expressing episodes, and they documented several occurrences of brief increased uterine activity during AME but no episodes of fetal tachsystole or uterine hyperstimulation. They also found no differences in gestational age at birth, NICU admissions, Apgar scores, birthweight, or delivery type between those assigned to AME and the control group [20]. Pilot research conducted by the DAME researchers [33] and a retrospective cohort study involving 94 women with diabetes, all of whom had been advised to engage in AME beginning at 36 weeks of gestation (16 who engaged in AME) [39], found that AME was associated with younger gestational age at birth and increased rate of neonatal admission to special care units/NICUs. Wide confidence intervals in both studies suggested that the observed differences may have occurred due to chance [40].

While this pilot study was not powered to detect between group differences in lactation outcomes, studies conducted outside of the U.S. have observed small effects of AME on reducing early infant formula use. In the DAME Trial, participants allocated to the AME arm had an adjusted relative risk of 1.15 (95% CI: 1.02, 1.28) of exclusive breastmilk feeding for the first 24 h after birth compared to the control group. More DAME participants assigned to AME were also exclusively breastfeeding during the birth hospitalization (57% vs. 49%) and at three months postpartum (60% vs. 55%), though these differences were not significant [20]. Similar to the DAME Trial, in a retrospective cohort study conducted at a public hospital in North Queensland, Australia and involving 357 women with diabetes in pregnancy, 80 of whom expressed milk in pregnancy, infants whose mothers engaged in AME were significantly less likely to receive formula during the birth hospitalization versus infants whose mothers did not engage in AME (OR 0.12, 95% CI: 0.05, 0.32) [21].

Rates of in-hospital infant formula supplementation in our study were high, regardless of group assignment (> 30%). Our previously published qualitative research from AME participant interviews conducted at 1–2 weeks postpartum provides some contextualization [19]. While participants reported that AME enhanced their prenatal and postpartum confidence that they would be able to breastfeed and make sufficient milk, similar to other studies on AME [17, 33], they also reported inconsistent and sometimes unsupportive policies at the birth hospital for antenatal milk storage and provision. Several participants were discouraged from using their antenatal milk when supplementation of direct chest/breastfeeding was advised. In addition, several AME participants experienced prolonged separation from their infant in the hospital due to birth complications and NICU admission. In these cases, the total volume of antenatal milk available was not always sufficient to avoid infant formula use altogether, particularly as the birth hospital at that time did not have a feeding policy to match supplemental feed volumes to physiologic need [41].

In the aforementioned qualitative findings from this pilot study, intervention participants felt that AME may have accelerated their milk coming in (i.e., onset of lactogenesis II) and contributed to abundant initial postpartum milk volumes [19]. While there is a lack of empirical support for this assertion and the current analysis was not powered to detect such relationships, there exists physiologic plausibility. It has been suggested that the period immediately proximal to birth represents a critical window during which milk expression/removal may drive up-regulation of prolactin receptors in breast tissue, thereby setting an increased threshold for long-term milk production and potentially attenuating the time to onset of lactogenesis II [42–44].

The optimal timing, “dose,” and format of AME education for various groups remains unknown. The DAME Trial and other studies on AME provided oral and/or written instructions for AME at 36 to 37 weeks of pregnancy, but not necessarily feedback on technique [25, 45, 46]. Casey et al. reported that all women in their retrospective cohort study in Australia were advised to begin AME at 34–36 weeks of pregnancy and were shown how to do AME by a midwife [21]. O’Sullivan et al. found that an online instructional video of AME evaluated among 95 pregnant, Australian women was effective in increasing participants’ knowledge and confidence in performing AME [47]. In regions where AME is not widely known or practiced, like the U.S., there may be a need to incorporate healthcare provider training and an evaluation of clinical/community infrastructures that may help to scaffold AME education. Our study used a “high touch” approach, where an IBCLC taught and reinforced AME in weekly face-to-face, one-on-one sessions, and we provided introductory education sessions to midwives at the recruitment site. Additional research is needed to understand how AME education might be tailored to optimize any potential lactation benefits, including proximal outcomes of breastfeeding self-efficacy and breastfeeding satisfaction, while accounting for scalability, costs, community needs, and cultural preferences. Our study team is currently conducting a large randomized trial of remote, telelactation-delivered AME education for nulliparous people in the U.S. with pre-pregnancy body mass indices ≥ 25, who are at risk for adverse lactation outcomes (PRenatal Video-Based Education and PostPARtum Effects (PREPARE Trial; NCT04258709)) [48].

In addition to the small sample size, there are several other limitations of this pilot trial. There was potential for bias in intervention delivery, in that the PI also functioned as the lactation consultant providing AME education. In addition, antenatal milk volumes were visually estimated by participants using a 5 mL anchor marking on the collection container; thus there was likely some degree of inaccuracy and imprecision in reported volumes. Wording of some survey items assessing feeding practices was imprecise, leaving open the possibility that we did not account for infants receiving shared or donor human milk, rather than their parent’s own milk. Finally, generalizability of findings is constrained by recruitment from a single midwifery practice, which resulted in a demographically homogeneous sample pool and enrolled participants who were mostly white and college-educated.

## Conclusions

These findings build upon our team’s previous qualitative research with the same study sample [19], collectively providing the first evidence of feasibility of AME among birthing people in the U.S. It is also the first investigation of AME among an exclusive group of nulliparous-to-primiparous individuals and those *without* diabetes. We found that weekly AME education and daily independent practice beginning at 37 weeks of pregnancy was feasible for this group. We also found no evidence that AME posed a safety risk for participants. AME provided a back-up supply of milk in several instances that likely reduced reliance on infant formula when supplementation was advised or desired. Findings require replication within a powered sample and with other populations at risk for poor lactation outcomes.

## Data Availability

The datasets generated during and/or analysed during the current study are not publicly available due to participants not consenting to public data sharing. However, data are available from the corresponding author on reasonable request.

## Abbreviations

AME: Antenatal milk expression
NICU: Neonatal intensive care unit
